# Supporting translation of research evidence into practice—the use of Normalisation Process Theory to assess and inform implementation within randomised controlled trials: a systematic review

**DOI:** 10.1186/s13012-023-01311-1

**Published:** 2023-10-27

**Authors:** Allison Williams, Laura Lennox, Matthew Harris, Grazia Antonacci

**Affiliations:** 1 Department of Primary Care and Public Health, Imperial College London, National Institute of Health Research (NIHR) Applied Research Collaboration (ARC) Northwest London, Charing Cross Campus, London, W6 8RP UK; 2 Business School, Centre for Health Economics and Policy Innovation (CHEPI), Imperial College London, Chelsea and Westminster Campus, London, SW10 9N UK; 3 Department of Primary Care and Public Health, Imperial College London, London, UK

**Keywords:** Implementation science, Randomised controlled trials, RCT, Normalisation Process Theory, NPT

## Abstract

**Background:**

The status of randomised controlled trials (RCTs) as the ‘gold standard’ for evaluating efficacy in healthcare interventions is increasingly debated among the research community, due to often insufficient consideration for implementation. Normalisation Process Theory (NPT), which focuses on the work required to embed processes into practice, offers a potentially useful framework for addressing these concerns. While the theory has been deployed in numerous RCTs to date, more work is needed to consolidate understanding of if, and how, NPT may aid implementation planning and processes within RCTs. Therefore, this review seeks to understand how NPT contributes to understanding the dynamics of implementation processes within RCTs. Specifically, this review will identify and characterise NPT operationalisation, benefits and reported challenges and limitations in RCTs.

**Methods:**

A qualitative systematic review with narrative synthesis of peer-reviewed journal articles from eight databases was conducted. Studies were eligible for inclusion if they reported sufficient detail on the use of NPT within RCTs in a healthcare domain. A pre-specified data extraction template was developed based on the research questions of this review. A narrative synthesis was performed to identify recurrent findings.

**Results:**

Searches identified 48 articles reporting 42 studies eligible for inclusion. Findings suggest that NPT is primarily operationalised prospectively during the data collection stage, with limited sub-construct utilisation overall. NPT is beneficial in understanding implementation processes by aiding the identification and analysis of key factors, such as understanding intervention fidelity in real-world settings. Nearly three-quarters of studies failed to report the challenges and limitations of utilising NPT, though coding difficulties and data falling outside the NPT framework are most common.

**Conclusions:**

NPT appears to be a consistent and generalisable framework for explaining the dynamics of implementation processes within RCTs. However, operationalisation of the theory to its full extent is necessary to improve its use in practice, as it is currently deployed in varying capacities. Recommendations for future research include investigation of NPT alongside other frameworks, as well as earlier operationalisation and greater use of NPT sub-constructs.

**Trial Registration:**

The protocol for this systematic review was accepted for public registration on PROSPERO (registration number: CRD42022345427) on 26 July 2022.

**Supplementary Information:**

The online version contains supplementary material available at 10.1186/s13012-023-01311-1.

Contributions to the literature

- This systematic review describes how NPT is used to assess and inform implementation within RCTs across diverse healthcare domains.
- By providing a comprehensive account of the application of NPT within RCTs, this review advances the Implementation Science literature, potentially contributing to the reduction of the research-implementation gap.
- The findings of this review facilitate informed decisions regarding the use of NPT as an appropriate theoretical approach to support RCTs and provide guidance for its utilisation within RCTs.

## Introduction 

### Background

The complexity of health systems often results in lengthy delays in the translation of research evidence into clinical practice. These delays impede improvements and jeopardise quality of care and patient safety [1, 2]. Studies have widely reported that, on average, it takes 17 years for research evidence to be implemented into daily clinical practice [3–5]. Delays can be partially attributed to the nature of the translation research pipeline, which encompasses several required processes each presenting opportunities for delay.

A study over a period of 17 years found that only 14% of clinical research was adopted into routine practice, contributing to the nearly 80% of clinical research funding that falls short of any meaningful public health impact [6, 7]. Implementation Science (IS) provides a promising avenue to appreciably reduce this lost potential through identifying determinants of implementation in various contexts and subsequently coalescing and implementing corresponding evidence-based strategies with the goal of increasing uptake [8–10].

Additionally, the uptake of interventions is often met with resistance when it disrupts established practices [11]. However, research suggests that incorporating evidence-based implementation strategies and qualitative methods to complement the quantitative nature of randomised controlled trials (RCTs) from the earliest phases of intervention development shows great potential for addressing these challenges and narrowing the research-implementation gap [12, 13].

Randomised controlled trials (RCTs) have ubiquitously been regarded as the ‘gold standard’ for evaluating the efficacy and safety of healthcare interventions, however this has become increasingly contested by the research community [14–18]. The large influx of RCT publications in recent decades [19, 20] poses challenges to healthcare systems to effectively manage the implementation of interventions at such volume [3].

### Potential for IS to Improve RCTs and Care

Revered for being uniquely qualified to draw objective cause-and-effect relationships, RCTs can achieve high internal validity on account of factors such as strictly controlled environments and standardised procedures [7, 13, 21]. They have a myriad of strengths including the randomisation of sample populations, prevention of extraneous variables and biases from influencing results and the ability to ensure adequate statistical power [13]. Limitations do exist, however, and the prioritisation of RCTs has been questioned on account of bias, ethical concerns and methodological and reporting errors [15–18, 22]. Further, RCTs can lack explanatory power on how to properly situate and implement the intervention into authentic practice settings [7]. This is in part due to strict inclusion criteria, and while these criteria strengthen the internal validity, they don’t always consider the heterogeneity of implementation in a real-world population [3, 7]. IS practices may ease this concern if used as a tool to consider and plan for the wider implementation of an intervention that is being tested in an RCT.

Merging RCTs with IS may provide significant benefit to the success of clinical research due to the role of RCTs in efficacy evaluations and the intentional planning and assessment of implementation processes that implementation research delivers [7]. As a note, for this review ‘implementation processes’ refers to any steps and factors that contribute to integrating and adopting an intervention. It is becoming more common for RCTs to employ an embedded or nested design, and process evaluations serve as a model example of how qualitative methods can support quantitative methods. These evaluations are adept at engendering participant and social perspectives of a trial and relaying measures of intervention fidelity, therefore augmenting insights [23–25]. Such insights may include whether the shortcomings of an intervention are rooted in the intervention itself, or the mechanisms of delivery and impact [26, 27]. Using process evaluations alongside RCTs is recommended by the Medical Research Council [23, 28], as it can assist in generalising interventions through unveiling the ‘black box’, and can be facilitated by the breath of theoretical approaches available to support translation [29, 30]. While there are a number of theoretical approaches which can aid in successfully adopting interventions into routine practice, Normalisation Process Theory (NPT) [31] is the most cited [32] and offers a promising framework for its capacity to conceptualise and explain implementation processes.

#### NPT

As a formal theory of action, NPT is intended to be utilised as a prospective explanatory framework and has been proposed as a suitable tool to narrow the research-implementation gap [33, 34]. NPT hones in on the actual work that stakeholders do that allow an intervention to be sustainably normalised into clinical practice. It is defined by May et al. [33] as:

‘A set of sociological tools to understand and explain the social processes through which new or modified practices of thinking, enacting and organising work are operationalised in healthcare and other institutional settings’ [35].^.^ p. 2

NPT postulates that embedding a new practice is enacted through the four constructs and sustained over time by stakeholders’ ongoing adherence to the processes of the practice [31, 36]. The constructs should be understood not as rigid chronological processes, but as dynamic and non-linear, as was intended by the original publication of the theory [33]. Definitions of the four NPT constructs adapted from Bracher et al. and Finch et al. [34, 37], and the associated sub-constructs, as defined by May et al. [38] are detailed in Table 1. The application of NPT can be seen in a diverse range of studies and settings. It has been used principally in qualitative implementation studies, as well as in studies such as complex healthcare interventions, technological interventions, feasibility studies and process evaluations [38–40]. NPT can also be used both prospectively, in the design of or during the intervention, and retrospectively, applied to data following the intervention, as well as with qualitative or quantitative data. There are also tools that facilitate various uses of NPT, for example, the NoMAD instrument [35, 36] is largely used to facilitate quantitative prospective use of NPT.

**Table 1:** NPT constructs and sub-constructs definitions as described in Bracher et al., Finch et al. and May et al. [34, 37, 38]

**Constructs**	**Coherence**	**Cognitive Participation**	**Collective Action**	**Reflexive Monitoring**
	The sense-making process and work that individuals and organisations do when they are faced with the problem of operationalising and routine embedding of a new practice	The relational process and work that individuals and organisations do to enrol, engage and sustain a community of practice around a new practice	The operational work that individuals and organisations do to implement the new practice	The informal and formal appraisal of implementation of a new practice to evaluate its advantages and disadvantages to promote embedding
**Sub-constructs**	**Differentiation**	**Initiation**	**Interactional Workability**	**Systematisation**
	Participants distinguish the intervention from current ways of working	Key individuals drive the intervention forward	Participants perform the tasks required by the intervention	Participants access information about the effects of the intervention
	**Communal Specification**	**Enrolment**	**Relational Integration**	**Communal Appraisal**
	Participants collectively agree about the purpose of the intervention	Participants agree that the intervention should be part of their work	Participants maintain their trust in each other’s work and expertise through the intervention	Participants collectively assess the intervention as worthwhile
	**Individual Specification**	**Legitimation**	**Skill-set Workability**	**Individual Appraisal**
	Participants individually understand what the intervention requires of them	Participants ‘buy in’ to the intervention	The work of the intervention is appropriately allocated to participants	Participants individually assess the intervention as worthwhile
	**Internalisation**	**Activation**	**Contextual Integration**	**Reconfiguration**
	Participants construct potential value of the intervention for their work	Participants continue to support the intervention	The intervention is adequately supported by its host organisation	Participants modify their work in response to their appraisal of the intervention

NPT has been used as a framework in numerous systematic reviews of various interventions [41–59], however few systematic reviews synthesise the literature on how NPT is operationalised in research [39, 60, 61]. The first systematic review, published in 2014 by McEvoy et al. [39] provided an early account of NPT and aimed to identify the interventions NPT was used in, how it was being operationalised and what the benefits were. It suggests future research to explore if NPT can shape the processes of implementation and result in increased embedding and integration of interventions. The second systematic review, published in 2018 by May et al. [60], aimed to qualitatively investigate the utilisation and limitations of NPT in mHealth implementation research of healthcare interventions, and to inquire into the role NPT plays in providing a deeper understanding of implementation processes. The review concludes that NPT is capable of explaining implementation processes correctly and that its flexibility allows for translation to a wide range of contexts [60]. The third systematic review, published in 2020 by Huddlestone et al. [61], is specific to primary care in the United Kingdom (UK) National Health Service (NHS) and aimed to identify which interventions utilise NPT in UK primary care, how it is being operationalised in these interventions and its acceptability among users. It concluded that, in the context of primary care, NPT offers an effective framework for understanding and explaining implementation processes and their associated challenges, notably in the management of chronic and comorbid health conditions [61].

NPT provides an intriguing lens for RCTs given its capacity to explore healthcare interventions from a different and more qualitative perspective, allowing for investigation into the work that is required for successful implementation of interventions once their efficacy is established through the standard clinical research pipeline. The relatively limited application of theoretically grounded qualitative methods used within RCTs [29, 30] contributes to the justification for this research. Further, previous systematic reviews do not explicitly focus on the use of NPT within the context of RCTs. For the purpose of this review, the phrase regarding the “use of NPT within RCTs” refers to the theory being used in relation to an RCT more generally and includes its use at any stage of the RCT, such as its use in a secondary follow-up study, in parallel to the main trial or as part of the initial trial design.

Therefore, this systematic review aims to examine the use of NPT to assess and inform implementation within RCTs by exploring how is NPT being operationalised within RCTs, what benefits are derived from the use of NPT and its contribution to understanding the dynamics of implementation processes within RCTs, and finally, what the challenges and limitations are of utilising NPT within RCTs.

## Methods

A qualitative systematic review of peer-reviewed literature was undertaken according to the Preferred Reporting Items for Systematic Reviews and Meta-Analyses (PRISMA) 2020 Statement [62], Additional file 1: Appendix 1.

### Searches

Eight databases (Embase, MEDLINE, APA PsycINFO, Global Health, Maternity & Infant Care Database (MIDIRS), Health Management Information Consortium (HMIC), Scopus and Web of Science) were preliminarily searched in May 2022 with the final search conducted on 6 June 2022.

Search terms included ‘Normalisation Process Model’ and ‘Normalisation Process Theory’, and were combined using the Boolean operator ‘OR’. The full search strategy can be viewed in Additional file 1: Appendix 2. Search terms such as ‘Extended Normalisation Process Theory’, or the abbreviations of the theory, were not used given that the chosen terms are within the name of the former, and that it is standard for the term to be written out before being abbreviated. Search terms relating to RCTs were not used as although the focus of this review is on the use of NPT within an RCT, some articles may report on the use in a separate article that was not itself an RCT. We found that these articles were not categorised as an RCT and would therefore not have been identified in a filtered search.

### Study Inclusion and Exclusion Criteria

Empirical peer-reviewed journal articles published in English language which discuss the use of NPT within the context of RCTs in a healthcare domain were considered for inclusion. Articles must have reported sufficient detail on the use of NPT, though there were not any restrictions regarding the healthcare topic, specialty, year of publication or geographic region. Exclusion criteria included wrong study design or setting, wrong use of NPT, such as inappropriate use of the theory for the purposes of this review or using its predecessor, Normalisation Process Model, non-English language, document type other than peer-reviewed empirical research journal articles, such as study protocols, conference proceedings or discussion papers, and any critical weakness found in their quality.

### Screening process

Following database searches, all citations were exported into EndNote 20 reference managing software and subsequently into Covidence [63], where the process of removing duplicate articles was automated and any missed duplications were removed manually. Using Covidence [63], the titles and abstracts of the remaining citations were screening by two authors. Full texts were then retrieved and reviewed, and included articles were subsequently moved to the next stage for quality assessment and data extraction.

### Study quality assessment

The Mixed Methods Appraisal Tool (MMAT), version 2018 [64] was used for the quality assessment of each article. While we only included articles which use NPT in an RCT, some articles reported on the NPT aspects of the RCT in a separate article that is not itself an RCT, for example qualitative studies and process evaluations. The MMAT permits for this variation and for all articles to be assessed using the same tool.

### Data extraction strategy

Rooted in the aim and objectives of this review, a data extraction template was developed and deployed in Covidence [63]. Extraction was completed by two authors and any discrepancies were resolved by the other authors to reach consensus. Data items to be extracted from each article included general identifying information, methodological items and a series of items specific to each of the three research questions. The full data extraction template can be found in Additional file 1: Appendix 3.

### Data synthesis and presentation

A narrative synthesis with tabular accompaniment was chosen due to the expected heterogeneity in methodology and resulting data [65], and was therefore deemed most appropriate to qualitatively analyse the data extracted from included articles. The elements which were performed include: (i) developing an initial descriptive synthesis to organise the extracted data from included studies to identify how NPT is being operationalised within RCTs, its reported benefits, challenges and limitations; (ii) exploring relationships in the data to consider factors that may provide insight into any observed differences in the benefits, challenges and limitations to implementation, and to understand how NPT can benefit implementation within RCTs; and (iii) assessing the robustness of the synthesis to determine the strength of the evidence for drawing conclusions about the benefits, challenges and limitations to operationalising NPT for implementation within RCTs identified in the synthesis and to determine the generalisability of the synthesis. Results are primarily presented narratively and are supported by figures and tabulation. Microsoft Excel was the primary tool used to identify common findings across the reviews as well as to create all Figures  [66].

### Protocol registration

The protocol for this systematic review was accepted for public registration on PROSPERO (registration number: CRD42022345427) on 26 July 2022 [67]. Details of the protocol can be accessed at: https://www.crd.york.ac.uk/prospero/display_record.php?ID=CRD42022345427.

## Results

**Fig. 1:**
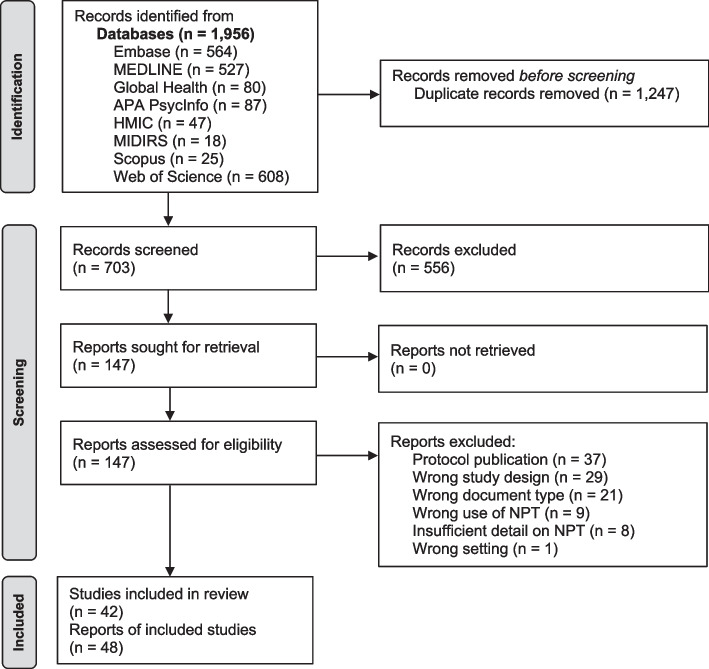
PRISMA flow diagram [62]

A total of 1,956 citations were identified and 48 articles reporting on 42 studies were eligible for inclusion. This process and the reasons for exclusion are illustrated in Fig. 1.

### Study quality assessment

Quality assessment found the studies to be of overall good quality. Accordingly, there were no exclusions on the grounds of quality or bias. Results of the quality assessment can be found in Additional file 1: Appendix 4. The robustness of the synthesis was also assessed by two authors who have academic and professional expertise in the field of IS by comparing results with other reviews on the application of NPT in various settings.

### General study characteristics

Included studies span from 2010 to 2022, and the range of countries, study design, methods and healthcare domain among included studies also do not vary widely. General study characteristics are summarised in Table 2. Most studies (55%) have been conducted in the UK followed by 19% in Canada, 10% in the USA, 5% in Australia and 2.4% in China, Denmark, Ireland, the Netherlands and South Africa. Of the 48 articles included, 54% (*n* = 26) are qualitative designs, 21% (*n* = 10) are mixed methods process evaluations, 15% (*n* = 7) are qualitative process evaluations, and 10% (*n* = 5) are either quantitative, mixed-methods, formative evaluation, cluster RCT (cRCT) or mixed-methods feasibility RCT designs. NPT has been applied within RCTs across a variety of healthcare settings.

**Table 2:** Characteristics of studies included in this systematic review

Study	Country	Study design	Healthcare setting	Healthcare topic	Application of NPT in RCT
1. Blickem et al. [68]	UK	Qualitative	Primary care	Chronic Kidney Disease Management	Prospective
2. Buckingham et al. [69]	UK	Mixed-methods feasibility RCT	Primary care	Palliative care, very severe COPD in older people	Prospective
3. Burridge et al. [70, 71]	Australia	Qualitative	Primary care	Complex chronic disease management–- type 2 diabetes	Prospective
4. Coupe et al. [72]	UK	Qualitative	Primary care	Collaborative Care for depression management	Prospective
5. Darley et al. [73]	UK	Mixed-methods process evaluation	Stroke services	Informal caregiver support	Prospective
6. Delvaux et al. [74]	UK	Qualitative	Stroke services	Arm recovery, rehabilitation after stroke	Prospective
7. Evans et al. [75]	UK	Qualitative	Primary care	Computer software prediction tool for emergency hospital admission risk	Prospective
8. French et al. [76]	UK	Qualitative process evaluation	Stroke services	Urinary incontinence after stroke	Prospective
9. Glidewell et al. [77]	UK	Qualitative process evaluation	Primary care	Adaptable evidence-based practice implementation package	Prospective
10. Glynn et al. [78]	Ireland	Qualitative	Primary care	mHealthh physical activity intervention implementation	Retrospective
11. Hassan et al. [79]	UK	Qualitative	Primary care	Severe mental illness in patients at risk of CVD	Retrospective
12. Hengel et al. [80]	Australia	Qualitative	Primary care	Sexual health CQI	Prospective
13. Hooker et al. [81–84]	Australia	Mixed-methods process evaluation / qualitative	Primary care	Family violence screening and care model	Prospective
14. Horwood et al. [85]	UK	Qualitative	Primary care	HCV vaccine uptake	Prospective
15. Hoskins et al. [86]	UK	Qualitative process evaluation	Primary care	Self-management of adult asthma	Prospective
16. Johnson et al. [87]	UK	Qualitative process evaluation	Acute hospital settings	Decision-making and communication in clinically unstable patients	Prospective
17. Keenan et al. [88]	UK	Mixed-methods process evaluation	Care facilities, older people	Dementia and challenging behaviour	Prospective
18. Kennedy et al. [89–91]	UK	Formative evaluation, Mixed-methods process evaluation	Primary care	Chronic condition self-care support	Prospective
19. Kousgaard et al. [92]	Denmark	Qualitative	Care facilities, older people	Overuse/inappropriate antibiotic prescribing for UTI	Retrospective
20. Lewis et al. [93]	UK	Mixed-methods process evaluation	Primary care	Domestic violence and abuse	Retrospective
21. Mackenzie et al. [94]	Australia	Qualitative	Primary care	Fall risk management	Prospective
22. Mäkelä et al. [95]	UK	Qualitative	Hospital at home, hospital	Geriatrician-led management of acute health events	Prospective
23. McInnes et al. [96]	Australia	Qualitative process evaluation	Emergency department	Evidence-based protocols for stroke management	Retrospective
24. Mishuris et al. [36]	USA	Quantitative	Primary care	Clinical prediction rules for sore throat and cough into EHRs	Prospective
25. Morden et al. [97]	UK	Qualitative	Primary care	Self-management support for osteoarthritis	Prospective
26. Morton et al. [98]	UK	Mixed-methods process evaluation	Primary care	Management of hypertension with digital interventions	Prospective
27. Myall et al. [99]	UK	Qualitative process evaluation	Primary cancer treatment	Self-management of cancer-related fatigue with digital interventions	Prospective
28. Nwolise et al. [100]	UK	Qualitative	Hospital	Advanced melanoma trial participation burden	Prospective
29. Ouyang et al. [101]	China	Mixed-methods process evaluation	Stroke services	Stroke, Acute Intracerebral Haemorrhage	Prospective
30. Patel et al. [102]	Australia	Mixed-methods process evaluation	Primary care	CVD management	Prospective
31. Saunders et al. [103]	UK	Qualitative	Primary care	Musculoskeletal pain	Retrospective
32. Schnabel et al. [104]	UK	Qualitative	Stroke services	Augmented arm rehabilitation, supported self-management after stroke	Prospective
33. Schubbe et al. [105]	USA	Qualitative	Surgery	Conversation aids for shared decision-making for breast cancer	Prospective
34. Sharpe et al. [106]	Canada	Qualitative	Primary care	Paediatric asthma	Retrospective
35. Spencer-Bonilla [107]	USA	Mixed-methods	Cardiology in Hospital	Shared decision-making for anticoagulation in atrial fibrillation	Prospective
36. Taft et al. [108]	Australia	Qualitative process evaluation	Primary care	Long-acting reversible contraceptives	Retrospective
37. Taylor et al. [109]	UK	Qualitative	Collaborative care	Depression in older people	Prospective
38. Valaitis et al. [110]	Canada	Qualitative	Primary care	Support for older adults, interprofessional teams	Prospective
39. Vest et al. [111]	USA	Qualitative	Primary care	Early stage chronic kidney disease	Prospective
40. Vos et al. [112]	Netherlands	Qualitative	Primary care	Colon cancer survivorship care	Prospective
41. Yapa et al. [113]	South Africa	Mixed-methods process evaluation	Primary care	Antenatal HIV care quality	Prospective
42. Yeung et al. [114]	Australia	Qualitative	Primary care	Chlamydia testing	Prospective

### Operationalisation of NPT

The operationalisation of NPT within the included studies is best categorised as either prospective, in which the initial utilisation of NPT occurs prior to or during the RCT, or retrospective, in which NPT is applied to data that had been previously collected during the RCT after its completion (Table 2). The majority of studies, 81% (*n* = 34) [36, 68–77, 80–91, 94, 95, 97–102, 104, 105, 107, 109–114], applied NPT prospectively. In exploring the relationship between retrospective or prospective use of NPT and the methodology used in the included studies, out of the 11 studies which used mixed-methods, six [69, 88–91, 98, 101, 113] applied NPT only to the qualitative aspects of the study and five [73, 81–84, 93, 102, 107] applied NPT both qualitatively and quantitatively, of which three studies [73, 93, 107] utilised the NoMAD tool. Of three studies that used the NoMAD tool, two [73, 107] were classified as prospective, which was to be expected given the intended use of the NoMAD tool, and one [93] was applied following the RCT and therefore classified as retrospective. To determine if the included studies enacted NPT in the way they intended to, protocols for the RCTs were also reviewed and the planned use of NPT was compared to how it was actually used. Among the 42 studies, protocols were found for 93% (*n* = 39), of which 31% (*n* = 12) mention NPT and subsequently operationalise the theory to, at minimum, the planned extent that was stated in the protocol.

### Theoretical coherence in NPT operationalisation

Though almost all included studies, 98% (*n* = 41), used each of the four constructs, the extent to which the sub-constructs of NPT were explicitly applied was less consistent. Only one study elected not to use all four constructs and omitted Reflexive Monitoring from their survey, stating that it would not be useful to collect appraisal since the survey was completed by participants prior to the implementation, and they would not have applicable knowledge to appraise the intervention at that point [107].

**Fig. 2:**
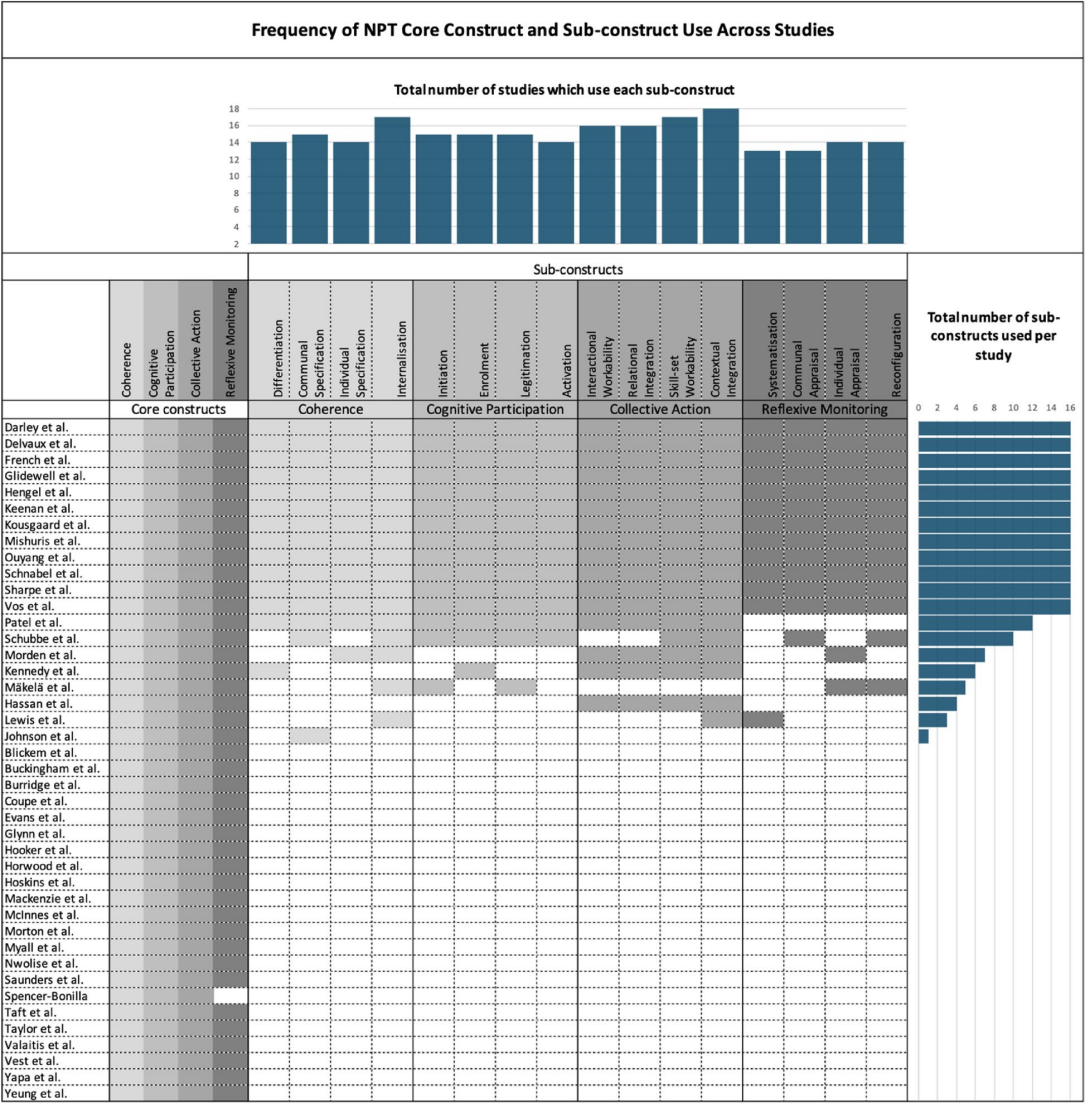
Frequency of NPT core constructs and sub-constructs used across included studies with totalled summaries

Regarding the operationalisation of the sub-constructs, while just over half of the studies (*n* = 22) [36, 68–72, 75, 78, 85–87, 95, 99–101, 103, 104, 108–111, 113, 114] did not specify the use of any sub-constructs, nearly 30% of studies (*n* = 12) [73, 74, 76, 77, 80–84, 89–91, 93, 97, 102, 105, 112] explicate their use of all 16 and use among the remaining studies (19%, *n* = 8) [79, 88, 92, 94, 96, 98, 106, 107] varied. Figure 2 further illustrates the frequency of sub-construct use. There were not any identifiable trends between omitted sub-constructs among the eight studies which chose to only use certain sub-constructs. However, the sub-constructs of Systematisation [79, 88, 92, 96, 98, 106, 107] and Communal Appraisal [79, 88, 92, 94, 96, 98, 107] were most frequently omitted, as seen in seven out of the eight studies, followed by Differentiation [79, 88, 94, 96, 98, 106], Individual Specification [79, 88, 92, 94, 96, 106], Activation [79, 88, 92, 94, 96, 98], Individual Appraisal [79, 88, 92, 94, 106, 107] and Reconfiguration [79, 88, 92, 94, 98, 107], each omitted by six out of the eight studies. There was an apparent lack of rationale among studies regarding their respective decisions to omit certain sub-constructs.

Across those included, although wording varied slightly, all studies appropriately defined the constructs in use, therefore demonstrating thorough understanding of the theory. However, four studies [70, 71, 73, 75, 107] appear to have misapplied the theory as was identified through operationalising the constructs in a linear manner, as further described in the below section.

### Consistency in NPT Operationalisation

Synthesis revealed consistency among studies in the overall operationalisation of NPT. Across all included studies, NPT is most commonly first operationalised during the data collection stage (52%, *n* = 22) [36, 69–71, 73, 74, 76–78, 87, 88, 92, 93, 96, 97, 104, 105, 107, 109, 110, 112–114], followed by data analysis (36%, *n* = 15) [68, 72, 75, 79, 80, 85, 86, 94, 95, 98, 100, 103, 106, 108, 111] and finally, from the outset of the design (12%, *n* = 5) [81–84, 89–91, 99, 102]. However, this should be interpreted with caution as it refers to the stage NPT is first deployed for each study and does not include subsequent utilisation.

**Fig. 3:**
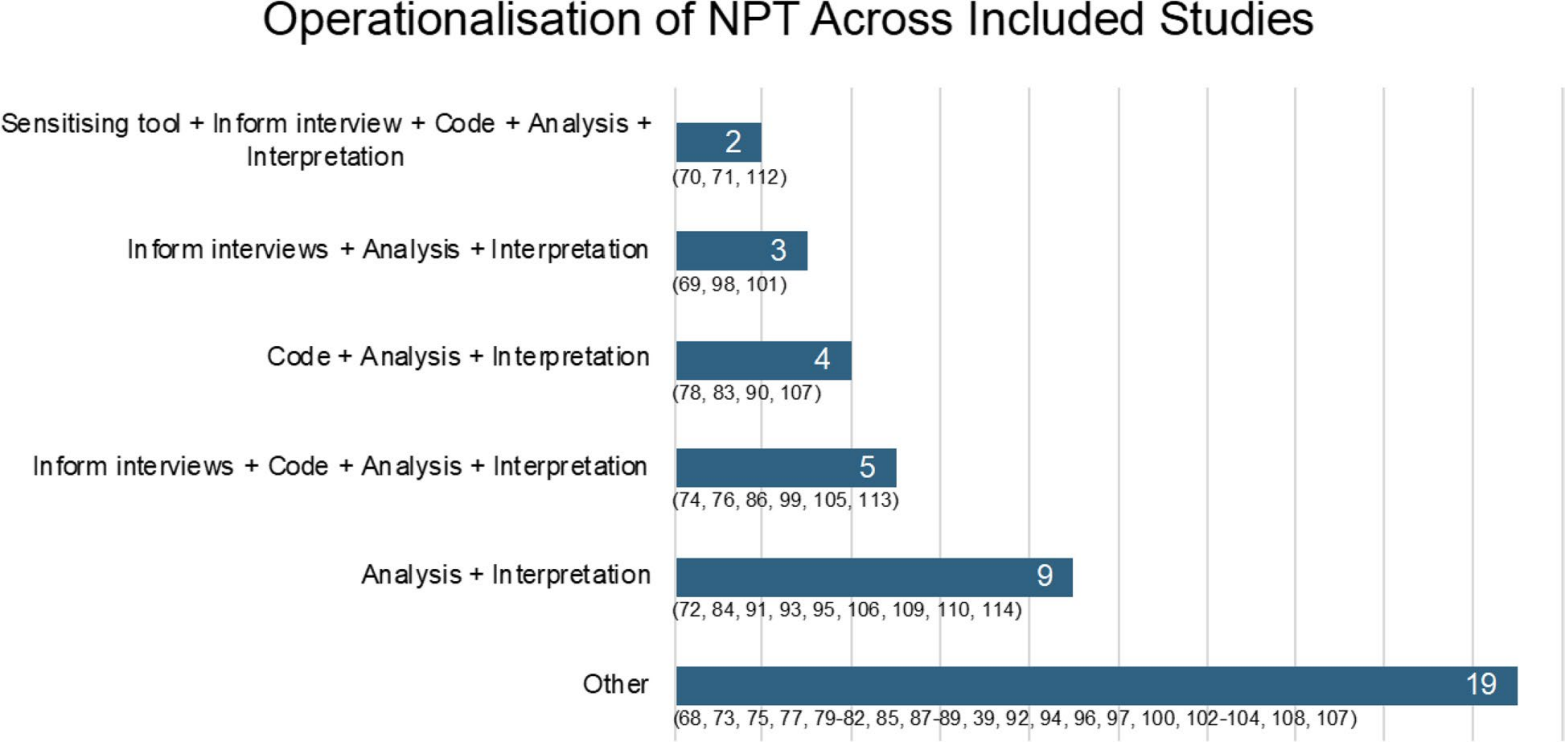
Number of studies per grouping of operationalisation

NPT was used for a total of 10 functions across the studies included in this review. One such function was to aid in the presentation and interpretation of results to some degree, which was seen in all studies. Additional functions of NPT that were seen among the studies include the following: in the development of the intervention being tested, development of a process evaluation, as a sensitising device, to inform interview topic guides, to guide focus groups, to inform survey development, to inform additional data collection methods such as observational data, field work and clinical note review, as a coding framework and finally, to analyse data. Figure 3 illustrates how the operationalisation of NPT was able to be classified into five groups for 23 of the studies due to their commonality in the way the theory was applied. The sixth ‘Other’ category represents the remaining 19 studies that a common theme of use could not be drawn. However, all studies operationalised NPT in at least one of the 10 functions as described above. As seen in Fig. 3, using NPT as a ‘sensitising tool’ refers to the theory guiding research to capture a specific perspective, ‘informing interviews’ refers to informing the development of the interview guides, ‘code’ refers to using NPT and its constructs as a coding framework to organise the data, ‘analysis’ refers to using NPT to identify key themes and ‘interpretation’ refers to using NPT as a lens for understanding the results.

**Fig. 4:**
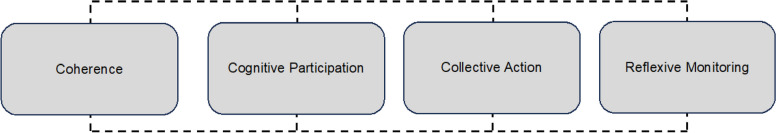
Example illustration adapted from Vos et al. [112] of the non-linear relationship between the NPT constructs

Further, only five studies [70–73, 75, 107] have drawn a type of causal or linear relationship between them other than the way the theory intends. For example, Spencer-Bonilla et al. [107] omitted the construct of Reflexive Monitoring on account that the intervention had not yet been regularly used, which may infer a perceived linear relationship where this construct is utilised last. Similarly, Evans et al. [75] refer to Coherence as the ‘first construct’ and explored it only prior to intervention implementation, whereas Cognitive Participation, Collective Action and Reflexive Monitoring were exclusively deployed in the analysis, which may also infer a linear relationship. A potential causal relationship can be inferred from Darley et al.’s [73] study in which it is stated that Legitimation, or ‘buy in’, relies on Enrolment from all stakeholders for an intervention to work. However, the remaining 90% (*n* = 38) of studies either do not infer causal or linear relationships between the constructs, or explicitly state otherwise using language such as ‘non-linear’, ‘dynamic’, ‘cyclical and on-going’, ‘interlinked’, ‘interrelated’, ‘operate simultaneously’ or as ‘generative mechanisms’ when referring to the relationships between constructs. Three studies [95, 102, 112] further demonstrate this non-linear and dynamic relationship between the constructs through illustrating their interconnectedness. An adapted example of this can be seen in Fig. 4.

Two studies provide critique in a way that can be understood as challenging the theoretical premise of NPT. Schubbe et al. [105] state that the theory does not allow for analysis of the patient perspective. Delvaux et al. [74] challenge the idea of NPT’s postulation that interventions are embedded in daily practice because of the work that people do, not what they believe.

### Benefits of NPT

Nearly all of the included studies reported on the benefits derived from the utilisation of NPT, with the exception of three studies [75, 95, 113] which did not. Extracted data suggests that NPT is viewed positively for use in the context of RCTs, and that NPT added value to deepening their understanding.

The most frequently reported benefits can be broken down into five main themes (Table 3). First, just over two-thirds of studies state that the theory improved their understanding of the dynamics of implementation processes (*n* = 28) [36, 68–71, 73, 76, 77, 79, 81–85, 87–91, 93, 96, 98, 99, 101, 102, 104–106, 108–112, 114], including its utility in understanding intervention fidelity within real-world settings, beyond simply determining whether or not objectives were achieved [102, 108]. To this point, Patel et al. found NPT to be useful in understanding intervention fidelity by providing an ‘explanatory focus through its emphasis on human agency’ [102], which allowed them to understand how implementation processes change over time across different settings and stakeholders. Further, Taft et al. found that using NPT for their analysis, including in analysing the fidelity checks which were conducted as part of the study, strengthened their understanding of sustainability [108]. Second, NPT provided depth to the understanding of the experiences of those involved in the intervention and what they perceived to be relevant and acceptable (*n* = 8) [68, 70, 71, 87, 89–91, 104, 105, 110, 112]. Third, NPT appears to be successful in its aim to understand and explain the work that is required, and who must be involved in the necessary processes, for interventions to be implemented, embedded and integrated into routine practice (*n* = 10) [31, 36, 68, 70, 71, 73, 77, 81–84, 89–91, 99, 102, 111]. Fourth, NPT is similarly reported to have provided an understanding of how effective, or not effective, an intervention is (*n* = 8) [36, 70, 71, 76, 81–84, 87, 103, 111, 114]. Finally, authors found NPT to allow for the analysis of multiple perspectives to provide a more holistic image of implementation practices [68, 72, 73, 77, 80, 98, 102, 104, 105, 109, 110, 112].

**Table 3:** The top five most reported benefits of utilising NPT with a direct quotation as a narrative example

Reported benefits of NPT	Example quotation
1. Understanding the dynamics of implementation processes (*n* = 28)	‘NPT provided an explanatory focus through its emphasis on human agency. By elucidating differences in implementation processes over time and between settings and various actors, we have been able to develop a nuanced understanding of intervention fidelity moving beyond whether it ‘worked’ or not’ [102] p 13
2. Identifying themes, key issues and factors that promote and inhibit implementation (*n* = 27)	‘For this study specific benefits were that NPT was used to generate the focus group and interview topic guides and to analyse the resulting dataset allowed us to identify factors that were likely to promote and inhibit the incorporation of this novel exercise promotion tool into an Irish primary health care environment’ [78]. p 8
3. Analysing data (*n* = 22)	‘The use of the four NPT constructs as an analytic framework enabled us to provide an understanding of how the AMBER care bundle did, and in many instances could not become normalised within an acute hospital setting’ [87] p 19
4. Identifying changes for future improvement and sustainable integration (*n* = 15)	‘The use of NPT in ACCEPt has also led to research in understanding sustainability in general practice and may be helpful for stakeholders in increasing effectiveness of implementing future interventions’ [114] p 6
5. Analysing multiple perspectives (*n* = 13)	‘Using NPT increased our understanding about how providers and patients individually saw the HT intervention in comparison to usual care in general and in relation to teamwork (Coherence), how the team collectively bought into the new model of care (Cognitive Participation), how providers put the intervention into action (Collective Action), and how providers and patients appraised it (Reflexive Monitoring)’ [110] p 11

Additional studies report their choice of NPT was justified on account of the constructs coinciding well with the intervention being implemented, as well as being a robust framework suitable to conceptualise the implementation of complex interventions with an intentional focus on contextual factors, such as dynamic social processes [75, 95, 113]. Authors reported that basing the analysis of implementation data on NPT increased the rigour of their analysis through providing a systematic, yet iterate method [68, 72, 78, 80–91, 93, 94, 96–99, 102, 108, 109, 111, 112, 114]. Appearing to result from the benefits cited of analysing data using NPT, a relatively consistent report emerged stating that NPT facilitated the identification of themes, key issues and factors which promote or inhibit implementation processes [36, 68, 69, 78–85, 87–92, 96, 101, 102, 104–112, 114].

Further benefits of NPT, though less widely reported, include its ability to identify specific changes and key areas of focus for future improvement and sustainability of implementation processes or an intervention itself (*n* = 15) [36, 69–71, 73, 76, 77, 81–85, 87–91, 98, 108, 111, 114]. NPT also provides an explanatory focus on outcomes, namely, the degree to which an intervention is normalised (*n* = 7) [74, 81–84, 87, 89–91, 96, 102, 103]. The flexibility of NPT was also reported to be of benefit to studies, given that it allowed for context-specific adaptations (*n* = 3) [36, 93, 105]. Benefits singularly reported include providing structure to discussions among the research team [89–91], useful visual representation of intervention strengths and weaknesses within each construct through radar plots developed by the NPT online toolkit [81–84], complimentary quantitative data produced by the NPT-informed NoMAD survey providing support to the interpretation of quantitative data [36] and lastly, deploying NPT throughout the lifecycle of the study was stated to minimise the risk of researcher bias [99].

### Challenges and limitations of NPT

In contrast to the benefits of NPT, there are significantly fewer reports on the challenges and limitations across included studies, with nearly 74% of studies (*n* = 31) [68–73, 75, 77, 78, 80, 85–87, 93–96, 98, 99, 101–104, 106–114] not delineating any challenges or limitations which emerged when utilising the theory.

The most frequently reported challenge relates to the overlap of data when coding or mapping themes back onto to the constructs (*n* = 4) [74, 79, 81–84, 92]. For example, Delvaux et al. found mapping interview transcript data onto the constructs ‘not straightforward’ due to the overlap between the constructs, and suggest that it should not be used alone on account that assessing the work without considering the beliefs and wider context is not sufficient to understand implementation [74], therefore challenging the theoretical premise of NPT. Addedly, Hassan et al. found the sub-constructs under Coherence, Cognitive Participation and Reflexive Monitoring to be unclear and could therefore not properly map themes due to unavoidable repetition [79]. Hooker et al. [81–84] found that applying NPT to empirical research, particularly to qualitative data, was challenging and required considerable investment to fully understand the complexities and dynamics of the constructs and sub-constructs and be able to operationalise the theory in pracitce [81–84]. Additionally, finding the NPT plots to be uninformative was also reported—ultimately resulting in the authors seeking an alternative visualisation method [36].

Limitations followed similar themes to the reported challenges, with the most commonly stated limitation being that NPT was not capable of addressing all aspects of the data collected, as shown in the case of Nwolise et al. [100] with regard to data on psychological burden (*n* = 4) [74, 76, 97, 100]. Contrasting findings also exist in terms of the diversity of perspectives NPT is able to consider. By contrast to the associated benefit previously stated, Delvaux et al. [74], Keenan et al. [88], Kennedy et al. [89–91] and Schubbe et al. [105] found the theory to lack the ability to capture multiple perspectives, as they state it was designed for perspectives of the health professionals implementing an intervention, hence requiring it to be adapted to include the patient perspective (*n* = 4) [74, 88–91, 105]. Additional limitations reported include the lack of validated absolute values derived from the radar plots of the NPT online toolkit, known as the NoMAD tool [35, 36], the construct’s inability to explain long-term implementation processes as well as NPT introducing inordinate influence prompting unintended consequences as a result [97]. For example, Morden et al. [97] found the constructs of NPT to influence and shift the focus of the study from data collection through to interpreting the findings due to potential themes falling outside the theory, which then must to be planned for and addressed to ensure all aspects of the study are considered with the same weight, regardless of whether or not they fit within the constructs of NPT [97].

## Discussion

### Main findings

This novel systematic review investigates the use of NPT to assess and inform implementation within RCTs in healthcare settings through systematically reviewing 48 articles reporting 42 studies. Overall, NPT is positively endorsed among researchers as it provides a useful theoretical framework for conceptualising and explaining the dynamics of implementation processes.

Across studies included in this review, NPT is primarily operationalised prospectively within RCTs, with the majority of studies first utilising the theory during the data collection phase, for example, to inform interview topic guides. This was also reflected in previous reviews with May et al. [60] and Huddlestone et al. [61] finding similar results. This prospective application is relative to each study’s associated RCT, which is noteworthy considering 95% of the studies were conducted alongside a trial rather than integrated into the design itself.

Regarding the constructs and sub-constructs of NPT, there is far less explicit utilisation of the sub-constructs than the four core constructs. It is unclear as to why full use of the sub-constructs was rather infrequent among included studies, with less than 30% operationalising all 16 sub-constructs, seeing as there is no justification stated by authors regarding the choice. Identification of the lack of sub-construct use across studies is unique to this review and has not been detailed in previous NPT reviews. Additionally, the majority of studies appropriately interpreting and applying the constructs, as well as understanding them to be non-linear and dynamic, provides evidence to suggest that NPT is theoretically coherent and is being operationalised with consistency in the context of RCTs.

Findings demonstrate that NPT is viewed positively by its users, a theme which emerged from the benefits reported in the included studies. Most frequently, NPT was noted as supporting understandings of the dynamics of implementation processes through highlighting aspects of intervention fidelity and the feasibility of implementation in real-world settings. This is done by aiding in the identification and analysis of key themes and issues which promote or inhibit such processes within RCTs. This finding is consistent with the findings from McEvoy et al. [39], May et al. [60] and Huddlestone et al. [61] which all report that NPT offers a suitable framework for accurately explaining complex implementation processes. Also often reported among included studies, and aligned with previous reviews [39, 60, 61], NPT successfully contributed to the ability of research teams to analyse implementation data guided by the NPT framework, and therefore allowing them to identify key themes and issues that promote or inhibit implementation processes. These are important findings and suggest that the theoretical framework is in fact able to explain implementation processes within RCTs despite being applied to varying degrees and across different stages of the research lifespan.

Challenges and limitations were scarcely reported across studies included in this review, even in the presence of reported benefits. The lack of critique and limited insight into NPT challenges has been noted in previous reviews [39, 60]. Among the one-quarter of studies in this review which did report on the challenges or limitations of utilising NPT within RCTs, it became evident that issues most often arose when coding and mapping data onto the constructs, as was similarly found in other reviews [39, 60]. Challenges centred around the theme of overlapping data presenting researchers with the choice of repeating data, or only placing it under one construct or sub-construct. Uncertainties such as this, coupled with difficulty in gaining a comprehensive understanding of the theory, may reduce the clarity and validity of outcomes sourced from the use of NPT. Several authors [74, 76, 97, 100] also had data fall outside the bounds of the NPT constructs.

### Strengths and limitations

To the best of our knowledge, this is the first systematic review to examine the use of NPT specifically within the context of RCTs. This systematic review extends our knowledge of NPT as a useful theoretical framework for both prospective and retrospective conceptualisation of factors essential to accelerating the continuum of implementation processes within RCTs, from informing their design to sustaining the incorporation of their interventions into routine clinical practice. This review has direct clinical relevance to the implementation of clinical interventions that are tested for efficacy in RCTs. In considering a primary goal of IS is to improve healthcare quality by increasing the adoption of research evidence into routine practice [115], there is also direct relevance to the field of IS in respect of the aims of this review.

Although this study has successfully demonstrated that NPT is a sound theoretical framework within the context of RCTs, there are limitations which must be considered. Considerations regarding possible reporting bias among included studies could impact the results of this review [116, 117], bearing in mind that the comprehensiveness of authors’ accounts of using NPT are relied on to inform the findings. This particularly concerns the lack of reported challenges and limitations and the varied level of detail in authors’ reflections on utilising NPT. Interpreting the findings of this review was challenging, given the lack of reporting of adverse experiences when using NPT across studies. In comparison with the 93% of studies that reflected on positive experiences of applying the theory, only 26% reported either a challenge or limitation. As a result, perception bias and potential reporting bias among included studies warrant caution when interpreting the weight of the positive reflections.

Considerations regarding the subjective nature of qualitative methods are essential as well. Perception bias due to individual perceptions and experiences are likely to influence discretionary choices regarding study selection, data extraction and synthesis [118]. A detailed account of the methodological approach and preventative measures, such as a predetermined data extraction template, contribute to making the process as objective as possible.

Publication bias may be present, since grey literature was not searched due to the inclusion criteria only considering published peer-reviewed journal articles [119]. While this was outside the scope of this review, future work may wish to include grey literature searches. In terms of transferability, limitations exist on account that the findings of this review are only applicable to RCTs within healthcare settings. The results cannot speak to the use of NPT in alternative study designs and contexts. However, this review adds to the existing literature on the theory given that NPT has shown similar benefits across multiple healthcare domains.

### Implications for future research and practice

Acknowledging that NPT is still a relatively new theory, especially within the realm of RCTs, more research is needed to determine its effectiveness at elucidating and informing implementation processes within RCTs. Further research to develop more straightforward guidelines on appropriately operationalising the theory could be useful, considering that the most noted difficulties reported by researchers relate to uncertainty. For example, uncertainty is reported around whether the theory could be appropriately applied to multiple perspectives beyond that of the professionals responsible for implementation [74, 88–91, 105]. The recent systematic review by May et al. [60] addressed this issue, yet studies as recent as Schubbe et al.’s [105] 2021 study understood it to focus on the perspective of health professionals and that it required adaptation to be applicable to the patient perspective.

Future research could assess how NPT compares to other theoretical frameworks, such as the Theory of Planned Behaviour (TPB) [120] or the Consolidated Framework For Implementation Research (CFIR) [121]. This review, as well as others [39], has found difficulties with data falling outside the constructs of NPT, such as the emotional motivations and intentions influencing implementation. The use of NPT in combination with other implementation science frameworks, such RE-AIM [122] and CFIR [121], could mitigate these shortcomings. Future work could explore how this may provide a more holistic understanding of implementation dynamics. For instance, a systematic review on CFIR applied with the Theoretical Domains Framework (TDF) for implementation research suggests that their combined use may better consider the intricacies of implementation processes [123].

With respect to future implications for NPT in practice, principal recommendations supported by the findings of this review call for earlier operationalisation of NPT, and greater use of its sub-constructs. A more in-depth plan on the proposed use of NPT starting from the protocol development would provide for a more rigorous and consistent use of the theory. Although the majority of studies applied the theory prospectively, only two [81–84, 89–91] first operationalised NPT from the outset of intervention design, which would allow for streamlining processes as a whole and potentially more effective and efficient implementation. Moreover, there was an evident lack of sub-construct use across included studies. These findings present missed opportunities for a deeper and more detailed understanding of the mechanisms of implementation processes.

## Conclusion

The findings of this systematic review describe how NPT is used to assess and inform implementation within RCTs across diverse healthcare domains. Results demonstrate that NPT is shown to be beneficial to RCT implementation processes and its use within RCTs has increased significantly, particularly over the past 3 years. Findings also highlight how NPT is being operationalised to support multiple functions including data analysis, interpretation and results presentation.

NPT appears to be especially beneficial in understanding of the dynamics of implementation processes through the identification and analysis of factors which play key roles in facilitating and impeding successful development, implementation and sustainability of interventions. Improving transparency concerning the challenges and limitations of utilising NPT is essential in determining the weight of these benefits.

Future work may wish to consider earlier and greater operationalisation of NPT, specifically in terms of its sub-constructs and the impact that deviation from the use of the theory in its entirety may have on its ability to improve implementation and, in turn, narrow the research-implementation gap.

### Supplementary Information

**Additional file 1: Appendix 1.** Adapted PRISMA [62] Checklist. **Appendix 2.** Systematic Search Strategy. **Appendix 3.** Data Extraction Template. **Appendix 4.** Study Quality Assessment – Adapted MMAT.

## Data Availability

The datasets used and/or analysed during the current study are available from the corresponding author on reasonable request.

## Abbreviations

IS: Implementation Science
RCT: Randomised controlled trial
NPT: Normalisation Process Theory
UK: United Kingdom
NHS: National Health Service
PRISMA: Preferred Reporting Items for Systematic Reviews and Meta-Analyses
cRCT: Cluster randomised controlled trial
CVD: Cardiovascular disease
CQI: Continuous quality improvement
HCV: Hepatitis C virus
mHealth: Mobile health
EHR: Electronic health record
COPD: Chronic obstructive pulmonary disease
UTI: Urinary tract infection
TPB: Theory of Planned Behaviour
CFIR: Consolidated Framework For Implementation Research
TDF: Theoretical Domains Framework
