# Supplementary material for: Supporting translation of research evidence into practice—the use of Normalisation Process Theory to assess and inform implementation within randomised controlled trials: a systematic review
Source: Implement Sci. 2023 Oct 27;18:55. doi: 10.1186/s13012-023-01311-1 (PMC10612208; doi:10.1186/s13012-023-01311-1)
Supplement: Supplementary file 1 — Additional file 1: Appendix 1. Adapted PRISMA [62] Checklist. Appendix 2. Systematic Search Strategy. Appendix 3. Data Extraction Template. Appendix 4. Study Quality Assessment – Adapted MMAT. [file 13012_2023_1311_MOESM1_ESM.docx]

# Appendices

## Appendix 1: Adapted PRISMA^62^ Checklist

| **Section and Topic** | **Item #** | **Checklist item** | **Page #** |
| --- | --- | --- | --- |
| **TITLE:** Supporting Translation of Research Evidence into Practice – the use of Normalisation Process Theory to Assess and Inform Implementation within Randomised Controlled Trials: A systematic review | | |  |
| Title | 1 | Identify the report as a systematic review. | Title page |
| **ABSTRACT** | | |  |
| Abstract | 2 | See the PRISMA 2020 for Abstracts checklist. | 2, 3 |
| **INTRODUCTION** | | |  |
| Rationale | 3 | Describe the rationale for the review in the context of existing knowledge. | 10 |
| Objectives | 4 | Provide an explicit statement of the objective(s) or question(s) the review addresses. | 10 |
| **METHODS** | | |  |
| Eligibility criteria | 5 | Specify the inclusion and exclusion criteria for the review and how studies were grouped for the syntheses. | 11 |
| Information sources | 6 | Specify all databases, registers, websites, organisations, reference lists and other sources searched or consulted to identify studies. Specify the date when each source was last searched or consulted. | 11 |
| Search strategy | 7 | Present the full search strategies for all databases, registers and websites, including any filters and limits used. | 11, 48, 49 |
| Selection process | 8 | Specify the methods used to decide whether a study met the inclusion criteria of the review, including how many reviewers screened each record and each report retrieved, whether they worked independently, and if applicable, details of automation tools used in the process. | 12 |
| Data collection process | 9 | Specify the methods used to collect data from reports, including how many reviewers collected data from each report, whether they worked independently, any processes for obtaining or confirming data from study investigators, and if applicable, details of automation tools used in the process. | 12 |
| Data items | 10a | List and define all outcomes for which data were sought. Specify whether all results that were compatible with each outcome domain in each study were sought (e.g. for all measures, time points, analyses), and if not, the methods used to decide which results to collect. | 12, 50, 51 |
|  | 10b | List and define all other variables for which data were sought (e.g. participant and intervention characteristics, funding sources). Describe any assumptions made about any missing or unclear information. | N/A |
| Study risk of bias assessment | 11 | Specify the methods used to assess risk of bias in the included studies, including details of the tool(s) used, how many reviewers assessed each study and whether they worked independently, and if applicable, details of automation tools used in the process. | 12 |
| Effect measures | 12 | Specify for each outcome the effect measure(s) (e.g. risk ratio, mean difference) used in the synthesis or presentation of results. | N/A |
| Synthesis methods | 13a | Describe the processes used to decide which studies were eligible for each synthesis (e.g. tabulating the study intervention characteristics and comparing against the planned groups for each synthesis (item #5)). | 12, 13 |
|  | 13b | Describe any methods required to prepare the data for presentation or synthesis, such as handling of missing summary statistics, or data conversions. | 12, 13 |
|  | 13c | Describe any methods used to tabulate or visually display results of individual studies and syntheses. | 12, 13 |
|  | 13d | Describe any methods used to synthesize results and provide a rationale for the choice(s). If meta-analysis was performed, describe the model(s), method(s) to identify the presence and extent of statistical heterogeneity, and software package(s) used. | 12, 13 |
|  | 13e | Describe any methods used to explore possible causes of heterogeneity among study results (e.g. subgroup analysis, meta-regression). | N/A |
|  | 13f | Describe any sensitivity analyses conducted to assess robustness of the synthesized results. | N/A |
| Reporting bias assessment | 14 | Describe any methods used to assess risk of bias due to missing results in a synthesis (arising from reporting biases). | N/A |
| Certainty assessment | 15 | Describe any methods used to assess certainty (or confidence) in the body of evidence for an outcome. | N/A |
| **RESULTS** | | |  |
| Study selection | 16a | Describe the results of the search and selection process, from the number of records identified in the search to the number of studies included in the review, ideally using a flow diagram. | 14 |
|  | 16b | Cite studies that might appear to meet the inclusion criteria, but which were excluded, and explain why they were excluded. | 14 |
| Study characteristics | 17 | Cite each included study and present its characteristics. | 14, 15, Table 2 |
| Risk of bias in studies | 18 | Present assessments of risk of bias for each included study. | 14, 52-55 |
| Results of individual studies | 19 | For all outcomes, present, for each study: (a) summary statistics for each group (where appropriate) and (b) an effect estimate and its precision (e.g. confidence/credible interval), ideally using structured tables or plots. | 14, 15, Table 2 |
| Results of syntheses | 20a | For each synthesis, briefly summarise the characteristics and risk of bias among contributing studies. | 15-24 |
|  | 20b | Present results of all statistical syntheses conducted. If meta-analysis was done, present for each the summary estimate and its precision (e.g. confidence/credible interval) and measures of statistical heterogeneity. If comparing groups, describe the direction of the effect. | N/A |
|  | 20c | Present results of all investigations of possible causes of heterogeneity among study results. | N/A |
|  | 20d | Present results of all sensitivity analyses conducted to assess the robustness of the synthesized results. | N/A |
| Reporting biases | 21 | Present assessments of risk of bias due to missing results (arising from reporting biases) for each synthesis assessed. | N/A |
| Certainty of evidence | 22 | Present assessments of certainty (or confidence) in the body of evidence for each outcome assessed. | 15-24 |
| **DISCUSSION** | | |  |
| Discussion | 23a | Provide a general interpretation of the results in the context of other evidence. | 25, 26 |
|  | 23b | Discuss any limitations of the evidence included in the review. | 25, 26 |
|  | 23c | Discuss any limitations of the review processes used. | 26, 27 |
|  | 23d | Discuss implications of the results for practice, policy, and future research. | 27, 28 |
| **OTHER INFORMATION** | | |  |
| Registration and protocol | 24a | Provide registration information for the review, including register name and registration number, or state that the review was not registered. | 3, 13 |
|  | 24b | Indicate where the review protocol can be accessed, or state that a protocol was not prepared. | 3, 13 |
|  | 24c | Describe and explain any amendments to information provided at registration or in the protocol. | N/A |
| Support | 25 | Describe sources of financial or non-financial support for the review, and the role of the funders or sponsors in the review. | 31, 32 |
| Competing interests | 26 | Declare any competing interests of review authors. | 31 |
| Availability of data, code and other materials | 27 | Report which of the following are publicly available and where they can be found: template data collection forms; data extracted from included studies; data used for all analyses; analytic code; any other materials used in the review. | N/A  (Though in the appendices: p. 48-55) |

## Appendix 2: Systematic Search Strategy

Embase Classic+Embase <1947 to 2022 June 07>

1 Normali?ation Process Model.mp. 25

2 Normali?ation Process Theory.mp. 541

3 1 or 2 564

Ovid MEDLINE(R) ALL <1946 to June 07, 2022>

1 Normali?ation Process Model.mp. 21

2 Normali?ation Process Theory.mp. 507

3 1 or 2 527

Global Health <1973 to 2022 Week 22>

1 Normali?ation Process Model.mp. 4

2 Normali?ation Process Theory.mp. 76

3 1 or 2 80

APA PsycInfo <1806 to May Week 5 2022>

1 Normali?ation Process Model.mp. 5

2 Normali?ation Process Theory.mp. 82

3 1 or 2 87

HMIC Health Management Information Consortium <1979 to March 2022>

1 Normali?ation Process Model.mp. 3

2 Normali?ation Process Theory.mp. 44

3 1 or 2 47

Maternity & Infant Care Database (MIDIRS) <1971 to May 24, 2022>

1 Normali?ation Process Model.mp. 1

2 Normali?ation Process Theory.mp. 17

3 1 or 2 18

Scopus – 25 results

TITLE-ABS-KEY ( "normalization process model" ) OR TITLE-ABS-KEY ( "normalization process thoery" )

Web of Science – 608 results

(TS=("normali?ation process model")) OR TS=("normali?ation process theory")

## Appendix 3: Data Extraction Template

| Data Extraction Items | Comments |
| --- | --- |
| **General Information:** |  |
| Title |  |
| First author |  |
| Year of publication |  |
| Journal |  |
| Study DOI |  |
| Country in which the study conducted |  |
|  |  |
| **Characteristics of Included Studies:** |  |
| Study design |  |
| Aim |  |
| Methods |  |
| Healthcare domain |  |
| Healthcare topic |  |
| Intervention design |  |
| Study limitations |  |
|  |  |
| **NPT** |  |
| Stage of study NPT was first operationalised |  |
| Description of stage NPT was first operationalised |  |
| How NPT was used within the study |  |
| Number of NPT core constructs used |  |
| Number of NPT sub-constructs used |  |
| Reported benefits of NPT |  |
| Reported challenges of NPT |  |
| Reported limitations of NPT |  |
| Recommendations for future research related to NPT |  |

## Appendix 4: Study Quality Assessment – Adapted MMAT

| 1. Qualitative Methodological Quality Criteria   (Answers options: Yes, No, Can’t Tell) | | | | | | | |
| --- | --- | --- | --- | --- | --- | --- | --- |
| Screening question 1. Are there clear research questions? | | | | | | | |
| Screening question 2. Do the collected data allow to address the research questions? | | | | | | | |
| - 1. Is the qualitative approach appropriate to answer the research question? | | | | | | | |
| - 1. Are the qualitative data collection methods adequate to address the research question? | | | | | | | |
| - 1. Are the findings adequately derived from the data? | | | | | | | |
| - 1. Is the interpretation of results sufficiently substantiated by data? | | | | | | | |
| - 1. Is there coherence between qualitative data sources, collection, analysis and interpretation? | | | | | | | |
| **Study** | **S1.** | **S2.** | **1.1.** | **1.2.** | **1.3.** | **1.4.** | **1.5.** |
| Blickem et al. | Yes | Yes | Yes | Yes | Yes | Yes | Yes |
| Buckingham et al. | Yes | Yes | Yes | Yes | Yes | Yes | Yes |
| Burridge et al. | Yes | Yes | Yes | Yes | Yes | Yes | Yes |
| Coupe et al. | Yes | Yes | Yes | Yes | Yes | Yes | Yes |
| Delvaux et al. | Yes | Yes | Yes | Yes | Yes | Yes | Yes |
| Evans et al. | Yes | Yes | Yes | Yes | Yes | Yes | Yes |
| French et al. | Yes | Yes | Yes | Yes | Yes | Yes | Yes |
| Glidewell et al. | Yes | Yes | Yes | Yes | Yes | Yes | Yes |
| Glynn et al. | Yes | Yes | Yes | Yes | Yes | Yes | Yes |
| Hassan et al. | Yes | Yes | Yes | Yes | Yes | Yes | Yes |
| Hengel et al. | Yes | Yes | Yes | Yes | Yes | Yes | Yes |
| Horwood et al. | Yes | Yes | Yes | Yes | Yes | Yes | Yes |
| Hoskins et al. | Yes | Yes | Yes | Yes | Yes | Yes | Yes |
| Keenan et al. | Yes | Yes | Yes | Yes | Yes | Yes | Yes |
| Kennedy et al. | Yes | Yes | Yes | Yes | Yes | Yes | Yes |
| Kousgaard et al. | Yes | Yes | Yes | Yes | Yes | Yes | Yes |
| Mäkelä et al. | Yes | Yes | Yes | Yes | Yes | Yes | Yes |
| McInnes et al. | Yes | Yes | Yes | Yes | Yes | Yes | Yes |
| Morden et al. | Yes | Yes | Yes | Yes | Yes | Yes | Yes |
| Myall et al. | Yes | Yes | Yes | Yes | Yes | Yes | Yes |
| Nwolise et al. | Yes | Yes | Yes | Yes | Yes | Yes | Yes |
| Saunders et al. | Yes | Yes | Yes | Yes | Yes | Yes | Yes |
| Schnabel et al. | Yes | Yes | Yes | Yes | Yes | Yes | Yes |
| Schubbe et al. | Yes | Yes | Yes | Yes | Yes | Yes | Yes |
| Sharpe et al. | Yes | Yes | Yes | Yes | Yes | Yes | Yes |
| Taft et al. | Yes | Yes | Yes | Yes | Yes | Yes | Yes |
| Taylor et al. | Yes | Yes | Yes | Yes | Yes | Yes | Yes |
| Valaitis et al. | Yes | Yes | Yes | Yes | Yes | Yes | Yes |
| Vest et al. | Yes | Yes | Yes | Yes | Yes | Yes | Yes |
| Vos et al. | Yes | Yes | Yes | Yes | Yes | Yes | Yes |
| Yeung et al. | Yes | Yes | Yes | Yes | Yes | Yes | Yes |
| 1. **Quantitative Randomised Controlled Trials Methodological Quality Criteria**   (Answers options: Yes, No, Can’t Tell) | | | | | | | |
| Screening question 1. Are there clear research questions? | | | | | | | |
| Screening question 2. Do the collected data allow to address the research questions? | | | | | | | |
| - 1. Is randomization appropriately performed? | | | | | | | |
| - 1. Are the groups comparable at baseline? | | | | | | | |
| - 1. Are there complete outcome data? | | | | | | | |
| - 1. Are outcome assessors blinded to the intervention provided? | | | | | | | |
| - 1. Did the participants adhere to the assigned intervention? | | | | | | | |
| Study | S1. | S2. | 2.1. | 2.2. | 2.3. | 2.4. | 2.5. |
| Buckingham et al. | Yes | Yes | Yes | Yes | Yes | No | Yes |
| Hooker et al. | Yes | Yes | Yes | Yes | No | Yes | Yes |
| Hoskins et al. | Yes | Yes | Yes | Yes | No | Yes | Yes |
| 1. **Quantitative Non-Randomised Methodological Quality Criteria**   (Answers options: Yes, No, Can’t Tell) | | | | | | | |
| Screening question 1. Are there clear research questions? | | | | | | | |
| Screening question 2. Do the collected data allow to address the research questions? | | | | | | | |
| - 1. Are the participants representative of the target population? | | | | | | | |
| - 1. Are measurements appropriate regarding both the outcome and intervention (or exposure)? | | | | | | | |
| - 1. Are there complete outcome data? | | | | | | | |
| - 1. Are the confounders accounted for in the design and analysis? | | | | | | | |
| - 1. During the study period, is the intervention administered (or exposure occurred) as intended? | | | | | | | |
| Study | S1. | S2. | 3.1. | 3.2. | 3.3. | 3.4. | 3.5. |
| N/A |  |  |  |  |  |  |  |
| 1. **Quantitative Descriptive Methodological Quality Criteria**   (Answers options: Yes, No, Can’t Tell) | | | | | | | |
| Screening question 1. Are there clear research questions? | | | | | | | |
| Screening question 2. Do the collected data allow to address the research questions? | | | | | | | |
| - 1. Is the sampling strategy relevant to address the research question? | | | | | | | |
| - 1. Is the sample representative of the target population? | | | | | | | |
| - 1. Are the measurements appropriate? | | | | | | | |
| - 1. Is the risk of nonresponse bias low? | | | | | | | |
| - 1. Is the statistical analysis appropriate to answer the research question? | | | | | | | |
| Study | S1. | S2. | 4.1. | 4.2. | 4.3. | 4.4. | 4.5. |
| Mishuris et al. | Yes | Yes | Yes | Yes | Yes | Yes | Yes |
| 1. **Quantitative Descriptive Methodological Quality Criteria**   (Answers options: Yes, No, Can’t Tell) | | | | | | | |
| Screening question 1. Are there clear research questions? | | | | | | | |
| Screening question 2. Do the collected data allow to address the research questions? | | | | | | | |
| - 1. Is there an adequate rationale for using a mixed methods design to address the research question? | | | | | | | |
| - 1. Are the different components of the study effectively integrated to answer the research question? | | | | | | | |
| - 1. Are the outputs of the integration of qualitative and quantitative components adequately interpreted? | | | | | | | |
| - 1. Are divergences and inconsistencies between quantitative and qualitative results adequately addressed? | | | | | | | |
| - 1. Do the different components of the study adhere to the quality criteria of each tradition of the methods involved? | | | | | | | |
| Study | S1. | S2. | 5.1. | 5.2. | 5.3. | 5.4. | 5.5. |
| Buckingham et al. | Yes | Yes | Yes | Yes | Yes | Yes | Yes |
| Darley et al. | Yes | Yes | Yes | Yes | Yes | Yes | Yes |
| Hooker et al. | Yes | Yes | Yes | Yes | Yes | Yes | Yes |
| Hoskins et al. | Yes | Yes | Yes | Yes | Yes | Yes | Yes |
| Johnson et al. | Yes | Yes | Yes | Yes | Yes | Yes | Yes |
| Kennedy et al. | Yes | Yes | Yes | Yes | Yes | Yes | Yes |
| Lewis et al. | Yes | Yes | Yes | Yes | Yes | Yes | Yes |
| Morton et al. | Yes | Yes | Yes | Yes | Yes | Yes | Yes |
| Ouyang et al. | Yes | Yes | Yes | Yes | Yes | Yes | Yes |
| Patel et al. | Yes | Yes | Yes | Yes | Yes | Yes | Yes |
| Spencer-Bonilla et al. | Yes | Yes | Yes | Yes | Yes | Yes | Yes |
| Yapa et al. | Yes | Yes | Yes | Yes | Yes | Yes | Yes |
